# Glycemic control of adult patients with type 1 diabetes mellitus in Arabian Gulf Countries; PREDICT

**DOI:** 10.1186/s12902-022-00946-3

**Published:** 2022-01-31

**Authors:** Khadija Hafidh, Nabila Ahmed Abdella

**Affiliations:** 1 Department of Medicine, Rashid Hospital, Dubai Health Authority, Rashid Hospital, P.0.Box 4545, Dubai, UAE; 2 Diabetes Department, Mubarak Al Kabeer Hospital, Kuwait City, Kuwait

**Keywords:** T1DM, Diabetes, Control, Gulf, HbA1c, Hypoglycemia

## Abstract

**Background:**

Optimum glycemic control is necessary to reduce and even prevent the risk of micro- and macrovascular complications of type 1 diabetes mellitus (T1DM). The main aim of this study was to assess the prevalence of T1DM patients with adequate glycemic control in 4 Arabian Gulf countries.

**Methods:**

This study was a multicenter, observational, cross-sectional disease registry. Data were collected from adult T1DM patients who were treated with insulin within 6 months prior to the study visit.

**Results:**

Out of 241 patients whose data were eligible for primary endpoint analysis, 27.4% had adequate glycemic control (HbA1c < 7%). The patients’ age ranged from 18 to 64 years, and 53% were males. The mean (SD) duration of diabetes was 14.6 (9) years and the mean HbA1c was 8.11 (1.8) %. At the time of T1DM diagnosis, mean HbA1c was 10.7 (2.17) %. About 98% of the patients were normotensive and the lipid profile of patients was found to be optimal. The main variables associated with adequate glycemic control were low HbA1c at diagnosis (*P* < 0.001) and absence of a family history of diabetes (*P* = 0.002).

**Conclusions:**

We found that the glycemic control of T1DM adult patients in Kuwait, UAE, Oman and Bahrain is suboptimal. More efforts are necessary to pinpoint the causes of inadequate control in this population.

**Supplementary Information:**

The online version contains supplementary material available at 10.1186/s12902-022-00946-3.

## Background

Type 1 Diabetes Mellitus (T1DM) is a chronic autoimmune disease characterized by immune-mediated destruction of insulin-producing beta cells of the pancreas; its main precipitating factors are genetic susceptibility and environmental insults [1, 2].

In the Middle East and North Africa (MENA) region, nearly 12.8% of the adult population aged 20 to 79 years old (corresponding to nearly 55 million people) were living with diabetes in 2019. According to the International Diabetes Federation (IDF), this prevalence is predicted to increase by a staggering 96.5% to reach 16.7% (or 108 million people) in 2045 [3]. Anders Green estimated that T1DM was prevalent in 102,500 subjects in the Middle East in 1995 [1]. In 2019, the number of children and adolescents (i.e. those aged 19 years or less) with T1DM in the Middle East and North Africa (MENA) region was 149,400 [4].

Insulin from an outside source is indispensable for the survival of T1DM patients [1]. Hyperglycemia is largely associated with injury to the vascular system; microvascular complications such as diabetic retinopathy, nephropathy and neuropathy occur with small vascular injury, while macrovascular complications such as cardiovascular problems result from injury to the large blood vessels [5]. As a result, T1DM is associated with an increased risk of premature death compared with that in people without diabetes [1, 6]. To prevent acute and chronic complications of T1DM, therapy should be in the form of a regimen consisting of regular exercise and meal plans in addition to insulin injections (the foundation of therapy) and self-monitoring of blood glucose [7]. In fact, the risk of developing diabetes complications largely correlates with the level of long-term glycemic control [1].

The most recent standards of medical care in diabetes put forward by the American Diabetes Association (ADA) in 2017 recommended an HbA1c goal less than 7% in non-pregnant adults with diabetes [8]. However, multiple studies found that the majority of T1DM patients do not meet their glycemic target recommended by major diabetes organizations. The barriers to optimal intensification of insulin therapy are multifactorial and vary between patients; they include hypoglycemia, weight gain, fear of injection, regimen complexity, perception of benefits, costs, and impact of treatment on everyday life [9].

Compared to the extensive work conducted on Type 2 Diabetes Mellitus (T2DM), T1DM has gained less attention; especially in developing countries [10]. This study aimed to assess the prevalence of T1DM adult patients with optimal glycemic control (HbA1C < 7%) in Arabian Gulf countries, and to collect data on the possible obstacles that could be preventing T1DM patients in these countries from reaching optimal glycemic control.

## Methods

### Study population

Already-diagnosed T1DM patients of both genders who were 18 years or older (except for Kuwait, where the minimum age was 21 years) and who were being treated with insulin within 6 months prior to the study visit were eligible for inclusion. All patients signed an informed consent form prior to any study procedures. Patients with any other type of diabetes (T2DM, gestational, or secondary), pregnant and breast-feeding women, or patients who were concurrently participating in other clinical trials were excluded.

### Study design and data collection

This was a multicenter, cross-sectional, non-interventional disease registry. Thirteen centers (8 public hospitals and 5 private clinics) participated in the study. In the UAE, the centers were Rashid hospital, Aster Jubilee clinic, American Hospital, and Emirates Hospital. In Kuwait, the centers were Tiba Hospital, Dasman Diabetes Institute, Mubarak Al Kabeer Hospital, and Al-Naeem primary healthcare center. The centers in Bahrain were Salmaniya Medical Complex (SMC), Royal Bahrain Hospital, and Ahmed Ali Kanoo Public Health center. The centers in Oman were Sultan Qaboos University Hospital (SQUH), and Bakra primary healthcare center. All participating centers managed T1DM patients through endocrinologists and primary care physicians. Centers were selected for the study through convenience sampling. Due to the observational nature of this study, no assessments were mandated; data were only collected following routine clinical practice, and data requested for this study were determined based on medical practice in the participating countries. Data about socio-demographics and medical history (including diabetes history, complications of diabetes, and comorbidities) were collected. Data on prior and current insulin regimens, concomitant medications, and glycemic control were also recorded. Final database extraction was done on the 21st of February 2019. History and type of hypoglycemic events and access to patient’s education, − as per the physicians’ opinion- were collected. Physical examination was performed for all patients and vital signs were recorded.

### Study objectives

The primary objective of this study was assessing the prevalence of adequate glycemic control (HbA1c < 7%) in adult patients with T1DM in Arabian Gulf countries (namely Kuwait, United Arab Emirates (UAE), Bahrain and Oman). The secondary objectives were describing the clinical profile of these patients, their insulin regimens and diabetes medications, complications and comorbidities related to T1DM, and frequency and types of hypoglycemic episodes in the 2 months prior to the study. In addition, we aimed to identify the nature of insurance coverage and to observe the patients’ access to educational programs on diabetes.

### Statistical analysis

The number of patients included must allow estimation with a sufficient precision to assess the prevalence of glycemic control in these patients. Referring to previous studies performed in the gulf region, controlled glycemic level was achieved in 9.5% of patients diagnosed with T1DM. The minimal size was calculated using the following simple formula based on the estimation of an observed percentage of 10% with two-sided 95% Confidence Intervals (CIs):

$$N=\frac{Z^2\ P\ \left(1-P\right)}{D^2}$$

Where N = sample size, Z = Z statistic for a level of confidence (1.96 for a given 95% CI), P = expected proportion, and D = precision.

According to the given formula, at least 216 T1DM patients were required to estimate an observed percentage of 10% with an absolute precision of 4% and a 95% CI. Assuming that 15% of participating subjects would not be evaluable for the primary analysis due to missing values or unfulfilled inclusion/exclusion criteria; a minimum of 254 patients should be enrolled. Moreover with 254 patients, we were able to estimate with a precision rate ≤ 10% all other proportions allowing to describe the profile of patients living with T1D in Gulf countries.

All data summaries were based on a single analysis set that included all eligible patients enrolled in the study. In general, data were summarized using frequency and percentages for categorical variables with their 95% CI. Mean, median, standard deviation (SD), range, and 95% CI were used for continuous variables. All statistical tests were performed using two-tailed tests at a 5% level of significance. For any comparative analyses, Chi-square was used for all categorical variables, while T-test/ ANOVA test was used for continuous variables. Missing data or unknown responses were not counted in the percentages.

## Results

**Fig. 1:**
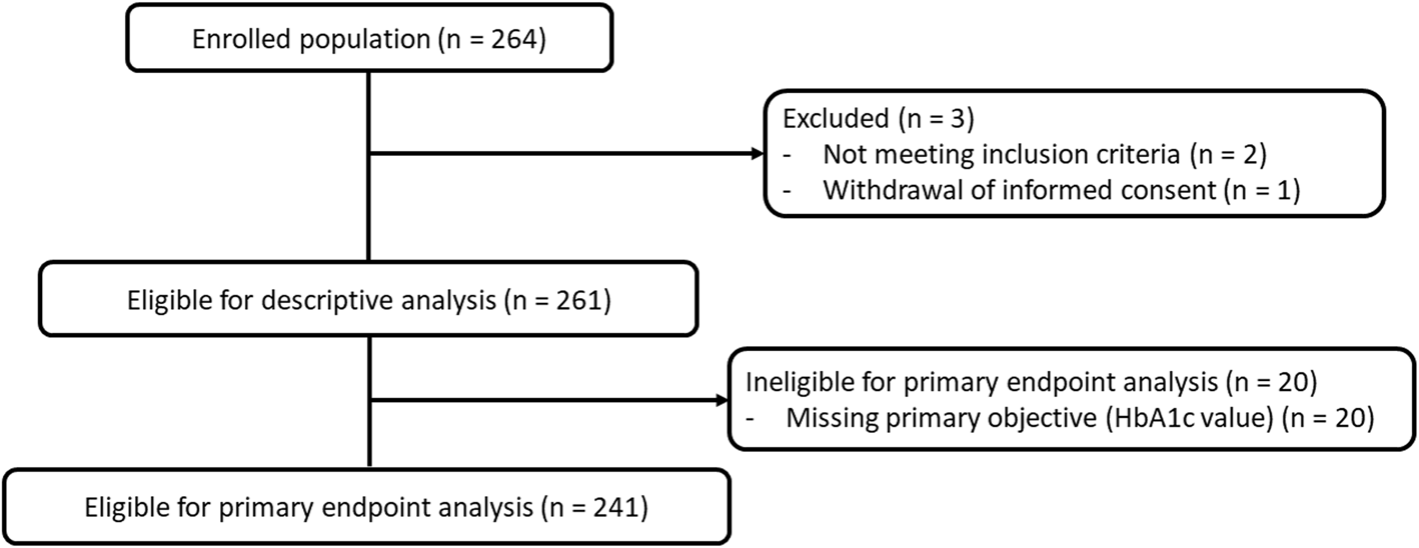
Patients’ Disposition

A total of 264 patients with T1DM were enrolled in this study; three patients were excluded and 261 were eligible for descriptive analysis. Of these, 20 patients lacked data on HbA1c values, resulting in a total of 241 patients being eligible for primary analysis. Figure 1 shows patients’ disposition in the study.

### Patients’ characteristics

The mean age (SD) of enrolled patients was 31.6 (9.4) years. The highest proportion of patients were from Kuwait (37.9%) and the least were from Oman (6.9%). The proportion of males was slightly higher than females (52.9% vs 47.1%). The highest level of attained education was graduate/post-graduate education at ~ 67%. The majority of patients (~ 96%) lived in urban areas and nearly 58% were full-time employed. Health insurance was provided to ~ 76% of the study population. Of these, nearly 57% had public health insurance. In addition, the most commonly prescribed concomitant medications were lipid-lowering drugs; all of which were statins. Detailed information on the characteristics of enrolled patients is in Table 1.

**Table 1:** Demographics and patients’ characteristics (*n* = 261)

Characteristic	
Mean age (SD) – years	31.6 (9.4)
Range	18–64
Mean duration of diabetes (SD) – years	14.6 (9.0)
Countries – count (%)	
Kuwait	99 (37.9)
UAE	77 (29.5)
Bahrain	67 (25.7)
Oman	18 (6.9)
Sex – count (%)	
Male	138 (52.9)
Female	123 (47.1)
Marital Status – count (%)	
Single	119 (45.9)
Married	137 (52.9)
Divorced/Widow	3 (1.2)
Missing	2 (0.8)
Education – count (%)	
None/Illiterate	1 (0.4)
Basic/Primary	10 (3.9)
Secondary	73 (28.6)
Graduate/Post-graduate	171 (67.1)
Missing	6 (2.3)
Residence – count (%)	
Rural	10 (3.8)
Urban	251 (96.2)
Employment – count (%)	
Full time	151 (57.9)
Part time	4 (1.5)
Unemployed	59 (22.6)
Retired	4 (1.5)
Unknown	43 (16.5)
Health Insurance – count (%)	
Present	199 (76.2)
Public Health Insurance	114 (43.7)
Private Health Insurance	70 (26.8)
Public & Private Health Insurance	15 (5.7)
Absent	62 (23.8)
Mean Height (SD) – cm	165.43 (9.9)
Males	173 (10.9)
Females	158 (8.7)
Mean Weight (SD) – Kg	71.69 (15.5)
Males	76.6 (12.5)
Females	66.1 (14.7)
Mean BMI^a^ (SD) – Kg/m^2^	26.2 (5.0)
Males	25.9 (5.0)
Females	26.6 (5.5)
BMI Range & Weight Status (*n* = 256) – count (%)	
< 18.5 (underweight)	7 (2.7)
18.5–24.9 (normal weight)	108 (42.2)
25.0–29.9 (overweight)	96 (37.5)
≥ 30 (obese)	45 (17.6)
Mean SBP^b^ (SD)– mmHg	121.2 (14.4)
Males	124.3 (14)
Females	117.7 (14)
Mean DBP^c^ (SD)– mmHg	73.8 (9.4)
Males	75.6 (8.9)
Females	71.91 (9.6)
Glycemic control	
Mean HbA1c^d^ (SD) – %	8.11 (1.8)
Males	7.9 (1.8)
Females	8.3 (1.7)
Mean FBG^e^ (SD) – mmol/L	9.6 (4.5)
Males	9.6 (4.3)
Females	9.6 (4.7)
Mean PPG^f^ (SD) – mmol/L	9.3 (4.5)
Males	9.6 (4.1)
Females	8.8 (5)
Smoking Status – no. (%)	
Never Smoker	211 (85)
Former Smoker	8 (3.1)
Current Smoker	31 (11.9)
Missing	1 (0.4)
Classes of Concomitant Medications – count (%)	
Antihypertensives	34 (13)
Antiplatelet Agents	12 (4.6)
Anticoagulants	1 (0.4)
Lipid Lowering Agents	65 (24.9)
Anticonvulsants	4 (1.5)
Thyroid medications	27 (10.3)
Vitamins / Multivitamins	45 (17.2)
Others	13 (5)

^a^ *BMI* Body Mass Index, ^b^ *SBP* Systolic Blood Pressure, ^c^ *DBP* Diastolic Blood Pressure, ^d^ *HbA1c* Glycated hemoglobin, ^e^ *FBG* Fasting Blood Glucose, ^f^ *PPG* Postprandial Glucose

### Primary endpoint analysis

We found that 27.4% (66 out of 241 patients) had adequate glycemic control. They were mainly from the UAE (44%), followed by Kuwait (30%), Bahrain (18%), and lastly Oman (8%).

### Secondary endpoints’ analyses

#### Hypoglycemia in the 2 months prior to study entry

During the 2 months preceding our study, five patients (1.9%) had severe hypoglycemia; two of which required hospitalization (Table 2).

**Table 2:** Incidence rate of hypoglycemia (*n* = 264)

Hypoglycemic Events^a^	Number of patients (%)
Probable Symptomatic hypoglycemia	111 (42)
Documented Symptomatic hypoglycemia	84 (31.8)
Severe hypoglycemia	5 (1.9)
• Required hospitalization	2 (0.8)

^a^Defined according to the American Diabetes Association workgroup report on hypoglycemia [11]

#### Clinical picture of T1DM at diagnosis

Nearly half of the patients in our study had a family history of diabetes. Clinical symptoms of the disease were evident at the time of diagnosis of approximately 89% of the patients. The main tests used in the management of T1DM were random blood sugar test (72.6%) followed by ketonuria (39%); while the least common were C-peptide test (1.2%) and glucose urine test (0.4%).

At diagnosis, most patients (nearly 98%) had normal blood pressure. The same proportion were not being prescribed any concomitant medications. The mean lipid profile was optimal. Mean HbA1C (SD) at diagnosis was high; 10.7 (2.17) %.

Detailed data on the clinical picture of T1DM at diagnosis are presented in Table 3.

**Table 3:** Clinical Picture at Diagnosis (*n* = 241)

Factors Pertaining to Clinical Picture at Diagnosis	
Family history of diabetes – no. (%)	
Yes	121 (50.2)
No	103 (42.7)
Unknown	17 (7.1)
Mean age at onset (SD)– yr	17.6 (9.0)
Methods of confirmed diagnosis* – no. (%)	
HbA1c^a^ test	74 (34.7)
Random blood sugar test	175 (72.6)
Fasting blood sugar test	54 (22.4)
Autoantibodies in blood	31 (12.9)
Ketonuria	94 (39)
Diabetic Ketoacidosis	21 (8.7)
C-peptide	3 (1.2)
Glucose urine test	1 (0.4)
Missing	1 (0.4)
Mean HbA1c at diagnosis (SD)– %	10.7 (2.17)
Lipid profile at diagnosis	
Mean total cholesterol (SD)– mmol/L	3.28 (0.9)
Mean HDL^b^ (SD)– mmol/L	5.1 (1.0)
Mean LDL^c^ (SD)– mmol/L	1.6 (0.9)
Mean Triglycerides (SD)– mmol/L	1.1 (0.4)
Blood pressure status at diagnosis	
Normotensive	215 (97.7)
Hypertensive	1 (0.5)
Hypotensive	4 (1.8)
Missing	21 (8.7)
Concomitant medications at diagnosis	
Yes	6 (2.5)
No	234 (97.5)
Missing	1 (0.4)
Clinical symptoms at diagnosis	
Yes	208 (88.5)
No	27 (11.5)
Missing	6 (2.5)

Note: Patients could have been diagnosed by more than one method

^a^ *HbA1c* Glycated Hemoglobin, ^b^ *HDL* High Density Lipoprotein, ^c^ *LDL* Low Density lipoprotein

#### Treatment of T1DM at the study visit

At the study visit, the majority (92 to 93%) were on basal-bolus insulin, while only 4.9% were on premix insulin. Basal insulin was mostly long-acting insulin analog (62%, 138 patients out of 222), while prandial insulin was mostly rapid-acting insulin analog (90.5%, 201 patients out of 222). In terms of off-label prescriptions for T1DM, metformin was the most commonly prescribed (8.3%, 20 out of 241 patients) compared to other antihyperglycemic medications. Further data on therapies received for T1DM at the study visit are in Table 4.

**Table 4:** Therapy with insulin and other antihyperglycemic medications at the time of visit (*n* = 241)

Antihyperglycemic Therapy	Count (%)
Basal Insulin	
Long-acting insulin analog	138 (57.3)
New generation of long-acting basal insulin	81 (33.6)
Intermediate-acting human insulin	3 (1.2)
Prandial Insulin	
Rapid-acting insulin analog	200 (83.0)
Short-acting human insulin	24 (10.0)
Premix Insulin	
Premixed analog insulin	10 (4.1)
Premixed human insulin	2 (0.8)
Other antihyperglycemic medications	
Metformin	20 (8.3)
DPP4^a^ inhibitors	7 (2.9)
SGLT2^b^ inhibitors	2 (0.8)
GLP1^c^ receptor agonists	3 (1.2)
Fixed dose combination of DPP4 inhibitor / Metformin	6 (2.5)

Note: Patient could have received more than 1 insulin type and more than 1 antihyperglycemic drug

^a^ *DPP4* dipeptidyl peptidase-4, ^b^ *SGLT2* sodium-glucose cotransporter-2, ^c^ *GLP1* glucagon-like peptide-1

#### Diabetes educational programs

Out of 241 patients, 130 patients (54%) previously participated in a diabetes educational program. These were largely conducted in hospital-based diabetes centers (87.7%) and were mainly delivered by certified diabetes educators (77.7%) and dieticians/nutritionists (71.5%). The main focus of these programs was on the correct use of medications (90%), on increasing awareness of the benefit of diet and exercise (85.4%), increasing skills on self-management (83.8%), increasing knowledge on the nature of diabetes (79.2%), and on the recognition and management of hypoglycemia (71.5%).

#### Comorbidities and Diabetes complications in T1DM patients

Out of 241 patients, 110 (45.6%) had comorbidities. The most commonly reported were hyperlipidemia (51.9%), hypertension (28.5%), hypothyroidism (15.5%), and obesity (13.6%). Diabetes complications were mainly diabetic retinopathy and neuropathy (each in 18.2% of patients), in addition to diabetic nephropathy (16.3%).

#### Variables associated with adequate glycemic control

To find out the factors that could potentially be associated with better glycemic control, we analyzed several characteristics among patients with adequate glycemic control (HbA1c < 7%) and inadequate glycemic control (HbA1c ≥ 7%).

Patients with adequate glycemic control were significantly older [mean age 34.1 (8.52) vs 31.4 (9.5); *P* = 0.046], had significantly lower body mass index (BMI) [25.2 (3.2) vs 26.7 (5.4); *P* = 0.009], significantly higher rate of probable symptomatic hypoglycemia in the 2 months prior to the study (51.5% vs 36.6%; *P* = 0.035), and significantly lower HbA1c at the time of T1DM diagnosis [9.6 (2.2) % vs 11.1 (2.0); *P* = 0.037]; compared to patients with inadequate glycemic control. Table 5 shows detailed data on the analyzed factors. When we conducted multivariate logistic regression for the variables, we found that HbA1c at the time of diagnosis and having a family history of diabetes; were significantly associated with adequate glycemic control (Supplementary Table 1).

**Table 5:** Variables associated with adequate glycemic control (HbA1c < 7%) in patients eligible for secondary analyses

Patient characteristics	Adequate Glycemic Control (HbA1c < 7%) (*n* = 66)			Inadequate Glycemic Control (HbA1c ≥ 7%) (*n* = 175)			*P*-value
	Valid ***n*** =	Count/ Mean	% / SD	Valid ***n*** =	Count/ Mean	% / SD	
Demographics and patients’ characteristics at the study visit							
Age – yr	66	34.1	8.5	175	31.4	9.5	0.046 ^a^
Female gender	66	28	42.4%	175	88	50.3%	0.276 ^b^
Health insurance present	66	53	80.3%	175	129	73.7%	0.289 ^b^
BMI - Kg/m^2^	64	25.2	3.2	173	26.7	5.4	0.009 ^a^
Duration of Diabetes – yr	63	15.4	10.4	169	14.4	8.43	0.491 ^a^
Comorbidities present	66	34	51.5%	175	76	43.4%	0.261 ^b^
Incidence rates of symptomatic hypoglycemia in the last 2 months							
Probable Hypoglycemia	66	34	51.5%	175	64	36.6%	0.035 ^b^
Documented Hypoglycemia	66	26	39.4%	175	48	27.4%	0.073 ^b^
At time of diagnosis							
Age at Onset – yr	63	19.1	10.7	169	17.1	8.3	0.172 ^a^
HbA1c - %	13	9.6	2.2	27	11.1	2.0	0.037 ^a^
Body weight – Kg	35	57.1	16.3	83	54.0	19.4	0.415 ^a^

^a^ Student t-test used to compare between subgroups among metric variables. ^b^ Chi-square test used to compare between subgroups among categorical variables. ^c^ Mann-Whitney U test used to compare between subgroups among non-parametric variables

When we subgrouped males and females into age categories, we found that the significant difference between adequate and inadequate glycemic control with older age was mainly apparent in females. Females who were ≥ 30 and < 40 years old were more adequately controlled (18.2% vs 16.6% inadequately controlled). This also applied to females who were ≥ 40 and < 50 years old (10.7% vs 4%); *P* = 0.028.

## Discussion

It has long been established that achieving optimal glycemic control in T1DM prevents and reduces the risk of developing microvascular and cardiovascular complications [12, 13]. Consequently, the ADA generally recommends an HbA1c target of less than 7% in non-pregnant adults and less than 7.5% in children and adolescents with T1DM [8, 14].

The best-case scenario of T1DM management is to extend the time of glycemic control while avoiding hypoglycemia and ketosis; or as described by the ADA workgroup “a lifetime of euglycemia without hypoglycemia” [11, 15]. In our study, less than third of our population (27.4%) were adequately controlled, with a mean HbA1c of 8.11% at the study visit (higher than the targeted HbA1c by an absolute value of 1.11%). Two months prior to study entry, 42% of the patients experienced probable symptomatic hypoglycemia (defined by the ADA workgroup as an event with typical symptoms of hypoglycemia which is not accompanied by determination of plasma glucose concentration) [11]. These numbers hint that the glycemic control of T1DM in Kuwait, UAE, Bahrain and Oman is sub-optimal. One reason for this might be the lack of self-monitoring of plasma glucose levels. This problem of suboptimal glycemic control is not unique to our region. Despite the advancements in T1DM technology over the past decade, suboptimal glycemic control remains a worldwide problem [16, 17]. In a recent global cross-sectional study by Renard et al., 24.3% of T1DM patients achieved HbA1c < 7%, with mean HbA1c being 7.95%. The lowest rate of glycemic control was in the Middle East (18.9%); with mean HbA1c being 8.21% which is very similar to the mean HbA1c observed in our study (8.11%) [18]. However, the rate of documented symptomatic hypoglycemia 2 months preceding our study was less than that reported for the Middle-Eastern population 3 months preceding the study by Renard et al. (31.8% vs 37.2%, respectively). Similarly, the rate of severe hypoglycemia in the previous 2 months to our study was less than that reported in the 6 months preceding the study by Renard et al. (1.9% vs 14%) [18]. A main contributing factor to these differences is the time in months during which hypoglycemia was measured.

In a cross-sectional survey of 17,000 Japanese patients with diabetes that aimed to describe glycemic control in Japan from 2000 to 2002, baseline characteristics of 793 T1DM patients were recorded. The mean HbA1c of T1DM patients in the Japanese study was 7.8% which is lower than that found in our study. In addition, the Japanese study population was older [mean age of 47 (15.8) vs 31.6 (9.4) years in our study], had lower BMI [22.4 (3.1) vs 26.2 (5.0) Kg/m^2^], similar systolic [124.5 (17.3) vs 121.2 (14.4) mmHg] and diastolic blood pressure [73.3 (10.2) vs 73.8 (9.4) mmHg], higher total serum cholesterol [199.6 (36.5) vs 126.8 (34.8) mg/dL] and higher level of triglycerides [101.3 (100.6) vs 97.4 (36.3)] [16].

Surprisingly, some patients in our study received other diabetes drugs (namely metformin, DPP4 inhibitors, SGLT2 inhibitors, and GLP1 receptor analogs) besides their insulin regimen. Although none of these medications is approved for use in T1DM, these were all prescribed “off-label”. The notion of adjunctive therapy in T1DM has been circulating in clinical practice for quite some time. Adjunctive therapies are usually used to reduce insulin dose requirements, contribute to more HbA1c reduction, and cause weight loss [19]; we speculate that they were used in this study for the same reasons. When metformin was added to insulin therapy in T1DM patients, small reductions in body weight, BMI, and lipid levels, as well as reduced insulin requirements were observed without improvements in HbA1c [20]. Furthermore, a systematic review and meta-analysis conducted by Wang et al. in 2018 found that the additional use of DPP4 inhibitors resulted in a greater (although not significant) reduction in HbA1c levels and a small reduction in postprandial glucose or insulin dose compared to insulin monotherapy. Despte this, Wang et al. did not support the addition of DPP4 inhibitors in real-life clinical management of T1DM [17]. Further, SGLT2 inhibitors in combination with insulin led to HbA1c improvement and body weight loss (but was associated with increased DKA risk) compared with insulin alone in inadequately controlled T1DM patients [21, 22]. Moreover, the addition of a GLP1 receptor agonist to insulin caused small HbA1c improvements as well as weight loss compared to insulin alone in T1DM patients [23].

Given that about 2.2% of our patients were neither on prandial nor premix insulin, it is worth mentioning that a small percentage of patients enrolled in this study could have been affected by latent autoimmune diabetes in adults (LADA). It is possible that they may have been registered as T1DM because they were positive for glutamic acid decarboxylase antibodies (GADA), but – due to their preserved beta cell function – did not necessarily require treatment with multiple daily insulin injections [24]. According to the study conducted by Maddaloni et al. in the UAE, 2.6% of 18,101 subjects with adult-onset diabetes had LADA [24]; a percentage approaching the 2.2% not receiving prandial or premix insulin in our study. O’Neal et al. highlighted that correct diagnosis and early treatment are crucial to evade long-term complications of poor glycemic control [25]. Therapy should aim to achieve glycemic control and preserve the ability of beta cells to secrete insulin [26]. An international expert panel concluded that DPP4 inhibitors or GLP1 receptor agonists may improve glycemic control in LADA patients (unless c-peptide levels are very low in the latter). It also suggested that SGLT2 inhibitors may be particularly promising in overweight LADA patients [26]. Considering that metformin was the most commonly prescribed medication among non-insulin antihyperglycemic drugs in our study, we should highlight that the mentioned expert panel concluded that the efficacy of metformin in LADA was inconclusive [26].

According to the recent position statement by the ADA, nearly 30% of T1DM in children and adolescents presents as diabetic ketoacidosis (DKA). However, DKA was employed in the diagnosis of only 8.7% of our study population. In our study, the most commonly used method for confirming diagnosis of T1DM was the random blood sugar test. The position statement also pointed out that the female to male ratio in children and adolescents with T1DM is usually 1:1; much similar to ours (it should be noted that our study did not include children but included adolescents and adults) [14].

Although several studies found a higher prevalence of dyslipidemia in children and adolescents with T1DM compared to controls [18, 27], the lipid profile of our study population at diagnosis of T1DM was optimal according to the goal levels suggested by Orchard et al. in 2001 [low-density lipoprotein (LDL) cholesterol < 100 mg/dL (2.6 mmol/L), high-density lipoprotein (HDL) cholesterol > 45 mg/dL (1.1 mmol/L), triglycerides < 150 mg/dL (1.7 mmol/L)] [19]. However, it should be emphasized that guidelines set by the European Society of Cardiology (ESC) and the European Atherosclerosis Society (EAS) for the management of dyslipidemias recommend reducing LDL cholesterol by at least 30% with statins in all T1DM patients with microalbuminuria and renal disease regardless the value of baseline LDL cholesterol [20]. In addition, most of our patients were normotensive.

Based on multivariate regression analysis, the main variables associated with adequate glycemic control in our study were low HbA1c at the time of diagnosis and absence of a family history of diabetes. A recent retrospective Jordanian study found that younger age, dietary compliance, receiving insulin at school, high grades in school, and the presence of direct mother care; were all associated with better metabolic control in T1DM Jordanian children [28].

Given that 96.2% of our study population resided in urban settings and 59.4% were employed, our results should not necessarily represent people with T1DM who are unemployed or those who live in rural areas. Given also that people who live in rural areas often face difficulties in accessing optimal healthcare and in adhering to the standard of diabetes care [21, 22], it should be expected that glycemic control in such population would be even less than that found in our study.

We believe that there are some obstacles opposing the uptake of educational programs (i.e. leading to poor attendance) in the region. Further studies are needed to determine whether these obstacles stem from clinicians not referring their patients to the programs or from patients not complying. Findings of such studies would be crucial to improve attendance in our hospitals.

The main strength of our study lies in the amount of data collected from T1DM patients in the participating centers. However, a few limitations exist. The exact names of insulin analogs and the duration and nature of diabetes educational programs were not recorded. In addition, the degree of adherence to insulin regimens and the QoL of patients were not assessed.

## Conclusion

The current study showed that the glycemic control of T1DM patients in Kuwait, Oman, UAE and Bahrain is still sub-optimal. Further studies are warranted to identify possible causes.

## Supplementary Information

**Additional file 1: Supplementary Table 1.** Multivariate Logistic regression analysis for predictors of adequate glycemic control (HbA1c of < 7%) in T1DM.

## Data Availability

The dataset supporting the conclusions of this article is available upon request from the corresponding author.

## Abbreviations

ADA: American Diabetes Association
BMI: Body Mass Index
CI: Confidence Interval
CRO: Clinical Research Organization
DBP: Diastolic Blood Pressure
DKA: Diabetic Ketoacidosis
DPP4: Dipeptidyl Peptidase-4
EAS: European Atherosclerosis Society
ESC: European Society of Cardiology
FBG: Fasting Blood Glucose
GADA: Glutamic Acid Decarboxylase Antibodies
GLP1: Glucagon-like Peptide-1
HbA1c: Glycated Hemoglobin
HDL: High Density Lipoprotein
IDF: International Diabetes Federation
LDL: Low Density Lipoprotein
MENA: Middle East and North Africa
PPG: Postprandial Glucose
QoL: Quality of Life
SBP: Systolic Blood Pressure
SD: Standard Deviation
SGLT2: Sodium-Glucose Cotransporter-2
T1DM: Type 1 Diabetes Mellitus
T2DM: Type 2 Diabetes Mellitus
UAE: United Arab Emirates
