# Supplementary material for: Glycemic control of adult patients with type 1 diabetes mellitus in Arabian Gulf Countries; PREDICT
Source: BMC Endocr Disord. 2022 Jan 31;22:32. doi: 10.1186/s12902-022-00946-3 (PMC8805329; doi:10.1186/s12902-022-00946-3)
Supplement: Supplementary file 1 — Additional file 1: Supplementary Table 1. Multivariate Logistic regression analysis for predictors of adequate glycemic control (HbA1c of < 7%) in T1DM. [file 12902_2022_946_MOESM1_ESM.docx]

## Supplementary Table 1. Multivariate Logistic regression analysis for predictors of adequate glycemic control (HbA1c of < 7% ) in T1DM

| **Variables** | **B** | **Wald.** | **P value** | **OR** | **95% CI** | |
| --- | --- | --- | --- | --- | --- | --- |
|  |  |  |  |  | **Lower** | **Upper** |
| **Age (years)** | 0.874 | 0.926 | 0.336 | 2.395 | 0.404 | 14.199 |
| **Gender (Females)** | 0.172 | 0.118 | 0.732 | 1.188 | 0.445 | 3.172 |
| **Health insurance (Yes)** | 0.191 | 0.192 | 0.662 | 1.210 | 0.515 | 2.844 |
| **Weight (cm)** | 0.102 | 0.714 | 0.398 | 1.107 | 0.874 | 1.403 |
| **Height (cm)** | -0.043- | 0.161 | 0.688 | 0.958 | 0.778 | 1.180 |
| **BMI (kg/m2)** | -0.366- | 1.187 | .276 | .694 | .359 | 1.339 |
| **DM Family history (Yes)** | -1.100- | 9.374 | 0.002 | 0.333 | 0.165 | .673 |
| **Age of Onset (Years)** | -0.905- | 0.992 | 0.319 | 0.404 | 0.068 | 2.401 |
| **Duration of Diabetes (Years)** | -0.829- | 0.830 | 0.362 | 0.436 | 0.073 | 2.596 |
| **HbA1c (%) at time of diagnosis** | -0.354- | 17.086 | 0.000 | 0.702 | 0.594 | .830 |
| **concomitant illnesses (Yes)** | 0.714 | 3.504 | 0.061 | 2.042 | 0.967 | 4.312 |
| **Probable Symptomatic hypoglycemia** | 0.471 | 1.076 | 0.300 | 1.602 | 0.658 | 3.900 |
| **Documented Symptomatic hypoglycemia** | -0.284- | 0.361 | 0.548 | 0.753 | 0.298 | 1.899 |
| **Severe hypoglycemia** | -20.847- | .000 | 0.999 | 0.000 | 0.000 | . |

*Notes: The logistic regression model was statistically significant, revealed* ***P <0.001,*** *the model showed prediction accuracy with* ***77.6%.*** *Results were confirmed by a sensitivity analysis with imputed missing data.*
